# The Draft Genome Sequence of *Actinokineospora bangkokensis* 44EHW^T^ Reveals the Biosynthetic Pathway of the Antifungal Thailandin Compounds with Unusual Butylmalonyl-CoA Extender Units

**DOI:** 10.3390/molecules21111607

**Published:** 2016-11-23

**Authors:** Anja Greule, Bungonsiri Intra, Stephan Flemming, Marcel G. E. Rommel, Watanalai Panbangred, Andreas Bechthold

**Affiliations:** 1 Department of Pharmaceutical Biology and Biotechnology, Institute of Pharmaceutical Sciences, Albert-Ludwigs-University of Freiburg, Stefan-Meier-Straße 19, 79104 Freiburg, Germany; anja.greule@pharmazie.uni-freiburg.de (A.G.); stefan.flemming@gmx.de (S.F.); marcel.rommel@yahoo.de (M.G.E.R.); 2 Department of Biotechnology, Faculty of Science, Mahidol University, 272 Rama 6 Road, Bangkok 10400, Thailand; aueng_i@hotmail.com (B.I.); watanalai.pan@mahidol.ac.th (W.P.); 3 Mahidol University and Osaka University Collaborative Research Center for Bioscience and Biotechnology, Bangkok 10400, Thailand

**Keywords:** *Actinokineospora bangkokensis*, thailandin, polyene, biosynthetic gene cluster, butylmalonyl-CoA, draft genome sequence, genome mining

## Abstract

We report the draft genome sequence of *Actinokineospora bangkokensis* 44EHW^T^, the producer of the antifungal polyene compounds, thailandins A and B. The sequence contains 7.45 Mb, 74.1% GC content and 35 putative gene clusters for the biosynthesis of secondary metabolites. There are three gene clusters encoding large polyketide synthases of type I. Annotation of the ORF functions and targeted gene disruption enabled us to identify the cluster for thailandin biosynthesis. We propose a plausible biosynthetic pathway for thailandin, where the unusual butylmalonyl-CoA extender unit is incorporated and results in an untypical side chain.

## 1. Introduction

Next generation sequencing and genome mining are powerful and rapid technologies to identify the genetic potential of a strain to synthesize secondary metabolites with various biological activities. One order known to produce many secondary metabolites with different bioactivities is the Actinomycetales. Under laboratory conditions only a few compounds are produced by a strain while their genomes comprise often more than 20 biosynthetic gene clusters. Cryptic clusters have been activated by heterologous expression [1], changing growth conditions [2] or by the manipulation of regulatory genes [3,4]. The knowledge of the genome sequence and the biosynthetic cluster composition of a secondary metabolite gives insights into the biosynthetic pathway. Therefore, it is a valuable tool for metabolic engineering to increase the production of a specific compound or to generate novel metabolites by combinatorial biosynthesis.

The genus *Actinokineospora* is a member of the order Actinomycetales and was introduced in 1988 as a separate genus [5]. The characteristics of this genus include having *meso*-diaminopimelic acid as component of their cell wall and the occurrence of menaquinone MK-9 (H4), phospholipids type II and *iso*-C_16:0_ fatty acids in their cell membrane. Until now only 16 strains of this genus have been identified. Thus, *Actinokineospora* belongs to the rare actinomycetes. Draft genome sequences are only available for *A. spheciospongiae* (GCA_000564855.1) [6], *A. enzanensis* (GCA_000374445.1) and *A. inagensis* (GCA_000482865.1).

The strain *Actinokineospora bangkokensis* 44EHW^T^ was isolated from the rhizosphere soil of an elephant ear plant (*Colocasia esculenta*) in Bangkok (Thailand) [7]. It produces thailandins A and B, antifungal polyenes with 28 membered macrocyclic lactone ring with two methyl groups, seven free hydroxyl groups and five conjugated double bonds. In addition, thailandin A is *O*-rhamnosylated at position C15, where thailandin B has only a hydroxyl group. Both compounds show significant inhibition of anthracnose fungi and pathogenic yeast strains [8].

In this study, we performed whole genome sequencing of *A. bangkokensis* 44EHW^T^ and successfully identified and verified the thailandin biosynthetic gene cluster. Herein, we report the putative biosynthetic pathway where the unusual butylmalonyl-CoA extender unit is incorporated into the polyketide chain.

## 2. Results

### 2.1. Draft Genome Sequence of Actinokineospora bangkokensis 44EHW^T^

**Figure 1:**
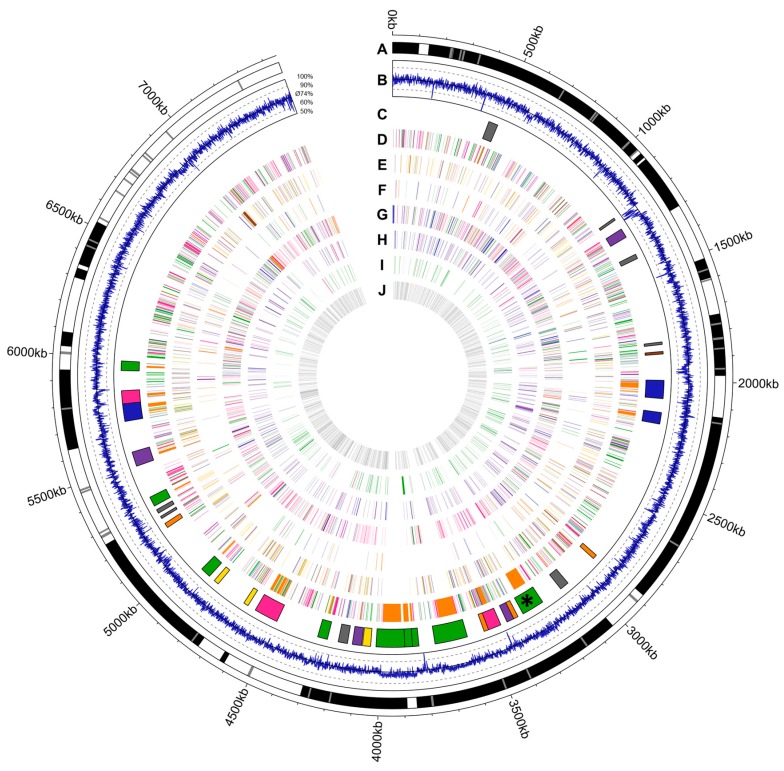
Illustration of *A. bangkokensis* 44EHW^T^ draft genome sequence. The genome has a size of 7.45 Mb. Circle A: Illustration of scaffold 1–32, shown in black and white, gaps are indicated in grey. Circle B: GC% content of 500 bp range, in 250 bp steps, between 50%–100%, line indicates average GC content of 74%. Circle C: Localization of putative secondary metabolite gene cluster, illustrated in PKS I (green), other PKS (purple), NRPS/PKS I (blue), other NRPS (pink), terpene (orange), siderophore (brown), lantipeptide (yellow) and other kind of cluster (grey); * Thailandin biosynthetic gene cluster is highlighted. Circle D: Localization of ORFs of general metabolism, subdivided into metabolism of amino acids (green), aromatic compounds (purple), fatty acids (blue), carbohydrates (pink), secondary metabolites (orange), and cofactors, vitamins and pigments (brown). Circle E: Localization of ORFs with putative modifying functions as carboxylases (green), dehydrogenases (purple), esterases (blue), hydratases (pink), hydrolases (orange), involved in redox reactions (brown), reductases (yellow) and transferases (grey). Circle F: Localization of ORFs putatively involved in ion metabolism, subdivided into metabolism of iron (green), phosphate (purple), sulfur (blue), nitrogen (pink) and other ions (orange). Circle G: Localization of ORFs putatively involved in replication/transcription/translation, subdivided into ORFs from nucleotide metabolism (green), protein-turnover and chaperons (purple), replication and repair (blue), transcription (pink), translation (orange) and tRNA metabolism (brown). Circle H: Localization of ORFs encoding putatively membrane proteins (green), transporters (purple), proteins involved in cell separation (blue) and from cell wall or membrane biosynthesis (pink). Circle I: Localization of ORFs putatively involved in defense and (stress-)response (green), from (pro-)phages (purple), for sporulation (blue) and communication (pink). Circle J: Localization of ORFs with unknown functions (grey).

A draft genome of *A. bangkokensis* 44EHW^T^ was sequenced and aligned to 32 scaffolds and 79 contigs. The largest scaffold has 931,456 nucleotides. The genome sequence consists of 7,453,713 nucleotides with an overall GC content of 74.1%, ranging from 44.2%–87.4% (calculated for 500 bp fragments). The sequence contains 6287 coding sequences (CDS), 50 tRNAs, and four clustered regularly interspaced short palindromic repeats (CRISPRs) predicted by the NCBI prokaryotic genome annotation pipeline [9]. The genome coding density is 87.9% and the average gene length is 1030 bp. This Whole Genome Shotgun project has been deposited at DDBJ/ENA/GenBank under the accession MKQR00000000. The version described in this paper is version MKQR01000000. An antiSMASH 3.0 [10] analysis revealed the presence of 35 gene clusters encoding secondary metabolite biosynthetic pathways (Table 1, Figure 1 and Appendix A, Table A1).

### 2.2. Identification and Verification of the Thailandin Biosynthetic Gene Cluster

Thailandins A and B were assumed to be synthesized by a polyketide synthase type I (PKS I). On the basis of the carbon chain length of the polyketide backbone, the PKS I is expected to have 14 modules. In the draft genome sequence of *A. bangkokensis*, three large PKS I clusters could be identified. Among these three clusters, cluster #11 encodes four PKSs comprising in total 14 modules. Cluster #16 has 20 modules, cluster #19 has at least 24 modules, yet it seems that the cluster is interrupted by the end of the scaffold. All three clusters have a higher GC content than the average genome. Cluster #11 has a GC content of 75.4%, cluster #16 and cluster #19 have 75.2% and 76.9%, respectively (Figure 1, circles B/C).

To verify that cluster #11 is responsible for thailandin biosynthesis, we interrupted the first PKS gene by a single crossover using a 3 kb internal fragment. Conjugation, electroporation as well as protoplast transformation was conducted for strain manipulation. However, only supplemention of MS plates with 10 mM CaCl_2_ could generate transconjugants, which were hygromycin resistant.

**Figure 2:**
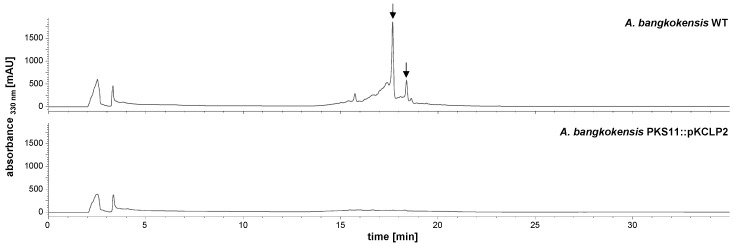
HPLC chromatogram of *A. bangkokensis* WT (**above**) and recombinant strain with interrupted PKS cluster #11 (**bellow**). Arrows indicate peaks of thailandin A (retention time 17.7 min) and thailandin B (retention time 18.3 min). The recombinant strain *A. bangkokensis* PKS11::pKCLP2 does not produce thailandins anymore.

The recombinant strain *A. bangkokensis* PKS11::pKCLP2 was tested for the production of thailandins in comparison to the wildtype strain. HPLC/MS analysis of organic extracts demonstrated that thailandins were not produced in the mutant strain (Figure 2). The result confirms that cluster #11 is the thailandin biosynthetic gene cluster.

**Figure 3:**
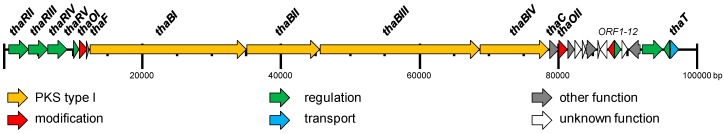
Genetic organization of the thailandin biosynthetic gene cluster in *A. bangkokensis* 44EHW^T^. The cluster spans a size of 96.1 kb with 25 open reading frames. Genes encoding the PKS I are yellow; genes encoding for modification are red; genes encoding for regulatory proteins are green; gene encoding a transporter is shown in blue; genes encoding other functions are grey; ORFs with unknown function are white.

The thailandin biosynthetic gene cluster was further analyzed in detail (Figure 3, Table 2). It spans 96.1 kb with 25 open reading frames (ORFs), but the main cluster probably contains only 13 genes (*thaRI*-*thaOII*, *thaT*). The polyketide synthase is encoded by the four genes *thaBI*, *thaBII*, *thaBIII* and *thaBIV*. Beside them, the cluster encodes a crotonyl-CoA carboxylase/reductase (*thaC*), two monooxygenases (*thaOI*, *thaOII*), seven regulatory genes (*thaRI*-*thaRIV; orf8/11/12*), one MFS transporter (*thaT*), and further genes with various functions. By BLAST analysis the function of five ORFs could not be assumed.

### 2.3. Proposed Thailandin Biosynthetic Pathway

**Figure 4:**
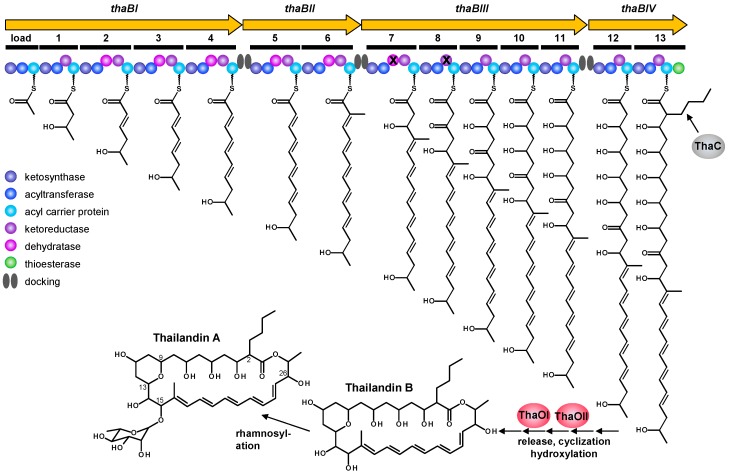
Putative thailandin biosynthetic pathway. The PKS encoding genes *thaBI*-*thaBIV* are shown in yellow; they encode for 14 modules (one loading module and 13 extender modules); the order of catalytic domains is shown in circles and color code (left), proposed X inactive domains; the single extender units are emphasized in bold. The crotonyl-CoA carboxylase/reductase ThaC is involved in butylmalonyl-CoA biosynthesis. The mature polyketide chain is released by the thioesterase domain of ThaBIV and cyclized. Furthermore, the polyene compound is hydroxylated by ThaOI or ThaOII and finally rhamnosylated, resulting in thailandin A.

Further analysis of the thailandin biosynthetic gene cluster led to the putative biosynthetic pathway of the compounds (Figure 4). The polyketide synthase is composed of one loading module and thirteen extender modules encoded by *thaBI*-*thaBIV*. They encode for domains of acyl carrier proteins (ACP), acyltransferases (AT), ketosynthases (KS), dehydratases (DH), ketoreductases (KR), docking (Dock) and a thioesterase (TE). The assembly line order is ThaBI [(KS-AT-ACP)-(KS-AT-KR-ACP)-(KS-AT-DH-KR-ACP)-(KS-AT-DH-KR-ACP)-(KS-AT-DH-KR-ACP)-Dock], ThaBII [Dock-(KS-AT-DH-KR-ACP)-(KS-AT-DH-KR-ACP)-Dock], ThaBIII [Dock-(KS-AT-DH-KR-ACP)-(KS-AT-KR-ACP)-(KS-AT-KR-ACP)-(KS-AT-KR-ACP)-(KS-AT-KR-ACP)-Dock] and ThaBIV [Dock-(KS-AT-KR-ACP)-(KS-AT-KR-ACP)-TE]. Futhermore, docking domains were identified between the PKS enzymes.

All acyltransferase domains have the typical GHSxG-Motif [11]. Except for the ATs of module 6 and 13, they all show specificity to the extender unit malonyl-CoA (x = LVIFAM). In the loading module, the AT domain has also malonyl-CoA-specificity. There, malonyl-CoA is probably decarboxylated by the KS of the loading module to provide an acetyl starter unit for transfer onto the first extension module. Like in other PKS systems with a KS domain in the loading module, the common cysteine of “condensing” KS domains is occupied by a glutamine in the active site [12].

The sequence of the AT domain of module 6 shows specificity to methylmalonyl-CoA (x = Q), which is consistent with the thailandin structure. In the last module, module 13, it is predicted that the extender unit ethylmalonyl-CoA is incorporated. In comparison with the polyene structure, we would suppose butylmalonyl-CoA as the unusual extender unit. Downstream of *thaBIV*, the gene *thaC* is located. The encoded protein is assumed to have a crotonyl-CoA carboxylase/reductase activity. It was shown, that crotonyl-CoA carboxylases/reductases are essential for the biosynthesis of various substituted malonyl-CoA extender units. They catalyze the NADPH-dependent carboxylation of α,β-unsaturated acyl-thioesters [13,14,15,16,17]. In the thailandin gene cluster, *thaC* encodes for such an enzyme, likely involved in the biosynthesis of butylmalonyl-CoA.

In module 7, a KS and a DH domain are located. Because of the hydroxyl group at C15 in the thailandin molecule, the DH domain is apparently inactive. This hydroxyl group is later used for the attachment of rhamnose moiety. The DH7 domain contains the conserved HxxxGxxxxP motif found in active DH domains, but possesses alterations of the GYxYGPxF, LPFxW, and Dxxx(Q/H) motifs [18].

After biosynthesis, the mature polyketide chain is released from the PKS and cyclized via the action of a thioesterase domain located at the C-terminal end of ThaBIV. The other cyclization takes place between C9 and C13. In other polyene biosynthetic pathways, this cyclization is formed by a keto and a hydroxyl group building a hemiketal ring [19,20]. Therefore, we assume that the KR domain of module 8 must be inactive, yet it contains all conserved amino acids of type A ketoreductases [21,22,23]. Following, the hydroxyl group could be transferred from C13 to C14 by an epoxide intermediate, which could be catalyzed by one of the two cytochrome P450 monooxygenases ThaOI or ThaOII. Alternatively, the tetrahydropyran ring could be generated by oxa-Michael addition on an α,β-unsaturated thioester intermediate. Therefore, special dehydratases and pyran-forming cyclases are required [24,25,26], which are not present within the cluster.

The other monooxygenase is likely responsible for hydroxylation of C26. For their activity, they require electrons from NADH, often mediated by ferredoxin. In the thailandin cluster, downstream of *thaOI*, the gene *thaF* is located, encoding for ferredoxin. Finally, thailandin B is rhamnosylated and results in thailandin A. Within the cluster no gene encodes for an enzyme with glycosyltransferase activity. A putative major faciliate transporter encoded near the structural genes, *thaT*, is suggested to be responsible for the transport of thailandin out of the producing organism. The other ORFs near the biosynthetic genes are unlikely to have an important role in the thailandin biosynthesis.

## 3. Discussion

*Actinokineospora bangkokensis* 44EHW^T^ produces the polyene compounds thailandin A and thailandin B [8]. Thailandin B is probably the precursor of the A-form, because thailandin A is further rhamnosylated. Both compounds show activity against pathogenic fungal strains with minimum inhibitory concentrations ranging between 16–32 μg/mL [8].

**Figure 5:**
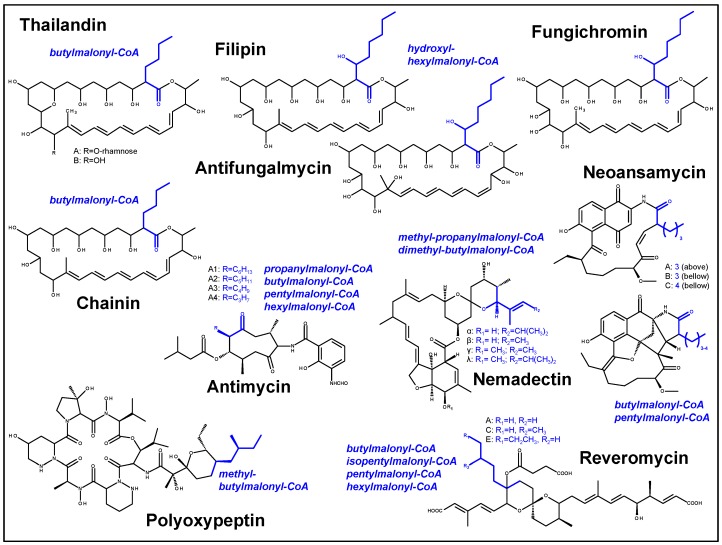
Examples for polyketides with unusual extender units. Unusual extender units are written in *italic*, shown blue in the chemical structure.

Polyene compounds are efficient antibiotics because they directly target the fungal plasma membrane by interacting with the main sterol, ergosterol, which often results in membrane permeabilization [27]. In addition, further acitivities of various polyene compounds have been demonstrated. The clinical application of these antifungal compounds is complicated by their low water-solubility and dose-dependent side effects, notably nephrotoxicity [28]. Many studies have been done to modify existing molecules in order to improve them. Thereby important structural elements were identified. Accordingly, the polyol [29] and polyene regions [30], the sugar modification, mainly by the aminoglycoside d-mycosamine [31,32], the exocyclic carboxyl group [30,33], and an additional aromatic heptaen side chain, which leads to haemolytic activity [34], seem to be particularly important for selective toxicity and activity. The modification or the loss of the D-mycosamine sugar moiety led to significant reduced antifungal activity [31,32]. Thailandin A has a rhamnose instead of d-mycosamine modification, but has also higher MIC compared to amphotericin B. Surprisingly, although thailandin B is not modified by a sugar moiety, it has even better antifungal activity than thailandin A [8]. In addition, both compounds do not have the exocyclic carboxyl group. In contrast to other polyenes, thailandins have an additional short side chain at C2 similar to chainin [35], filipin [36], fungichromin [37] and antifungalmycin [38] (Figure 5).

The genus *Actinokineospora* belong to the group of rare Actinomycetales with great potential to produce novel secondary metabolites. Only 16 strains of this genus are known to date. Many studies have been carried out to categorize these strains, yet studies into their secondary metabolite production is limited (Table A2). Recently, new antitrypanosomal and antioxidant compounds actinosporins were isolated from *A. spheciospongia* EG49^T^ [39,40]. Co-cultivation of this strain with *Nocardiopsis* sp. RV163 led to induction of further secondary metabolite biosynthesis [41]. Furthermore, only three other *Actinokineospora* genomes have been sequenced, *A. enzanensis* DSM 44649^T^ (ID 1120934) (8119858 bp, GC 70.8%, 37 predicted gene cluster), *A. inagensis* DSM 44258^T^ (ID 1120935) (7278759 bp, GC 70.2%, 34 predicted gene cluster) and *A. spheciospongia* EG49^T^ (ID 909613) (7529476 bp, GC 72.8%, 36 predicted gene cluster). In this study, we sequenced the genome of *A. bangkokensis* 44EHW^T^. The draft genome has 7.5 Mb with 74.1% GC content, which is significantly higher than the other *Actinokineospora* genomes. It is also remarkable, that there are many regions within the genome with a GC content <50%, as well as the three large PKS I gene clusters have an overall higher GC content of 75.4% (cluster #11, thailandin cluster), 75.2% (cluster #16) and 76.9% (cluster #19). This indicates a high frequeny of horizontal DNA transfer during evolution.

The antiSMASH analysis of the genome revealed 35 putative secondary metabolite gene clusters. The detailed evaluation of the PKS encoding clusters led to the assumption that cluster #11 should be responsible for thailandin biosynthesis. This hypothesis was proved by targeted inactivation of the first PKS gene *thaBI*. For genetic manipulation, different protocols were conducted without success. Finally, the supplementation of 10 mM CaCl_2_ resulted in the mutant strain via conjugation. The addition of CaCl_2_ could also increase conjugation frequency of several *Streptomyces* strains [42]. Noteworthy, *A. bangkokensis* is resistant against the commonly used antibiotics apramycin and spectinomycin.

The thailandin biosynthetic gene cluster encodes next to the PKS enzymes for proteins with regulatory function, post-polyketide modification, one transporter and few other proteins. Remarkably, there is no gene within the cluster which encodes a glycosyltransferase. In other polyene biosynthetic gene clusters, the genes for biosynthesis of the sugar moiety and the glycosyltransferase are present [43]. In the genome of *A. bangkokensis* 45 glycosyltransferase genes could be identified, of which two genes are in the PKS cluster #19 and nine in cluster #16, respectively. One of them should catalyze the rhamnosylation of the thailandin aglycon.

Beside modules 6 and 13, the bioinformatic analysis identified malonyl-CoA as extender unit, which corresponds to the chemical structure of thailandin. In module 6, methylmalonyl-CoA should be incorporated. In the last module we postulated the incorporation of butylmalonyl-CoA. The high diversity among polyketides is caused by the number of modules in the PKS assembly line, the presence of reducing domains, and other modifying enzymes, whereas malonyl-CoA, methylmalonyl-CoA or ethylmalonyl-CoA are usually incorporated. Therefore, butylmalonyl-CoA is an unusual extender unit. From structural analysis, we would also suppose the incorporation of butylmalonyl-CoA in chainin biosynthesis, but so far there is no cluster information available. The usage of unusual extender units is also postulated in few other biosynthetic pathways, but often the acyltransferases seem to be less specific, incorporating not only one defined unit. The AT domain of RevA of the reveromycin biosynthesis putatively uses butylmalonyl-CoA, isopentylmalonyl-CoA, pentylmalonyl-CoA or hexylmalonyl-CoA [44]. In the biosynthesis of neoansamycin A-C, pentylmalonyl-CoA or butylmalonyl-CoA is incorporated [45]. Different acylmalonyl-CoA extender units are proposed on the basis of various derivatives in both antimycin [46] and nemadectin [47]. In polyoxypeptin, methylbutylmalonyl-CoA is putatively used as an extender unit [48]. In the polyene compounds fungichromin, filipin and antifungalmycin, an unusual extender like hydroxyl- hexylmalonyl-CoA should be incorporated. These secondary products with their unusual extender units are shown in Figure 5. The incorporation of the lengthened extender unit in thailandin, chainin, filipin, fungichromin and antifungalmycin leads to the particular C2 side chain of the molecules, which is not common in other polyene compounds. Further structural studies would shed new light on polyene–fungus interaction.

**Figure A1:**
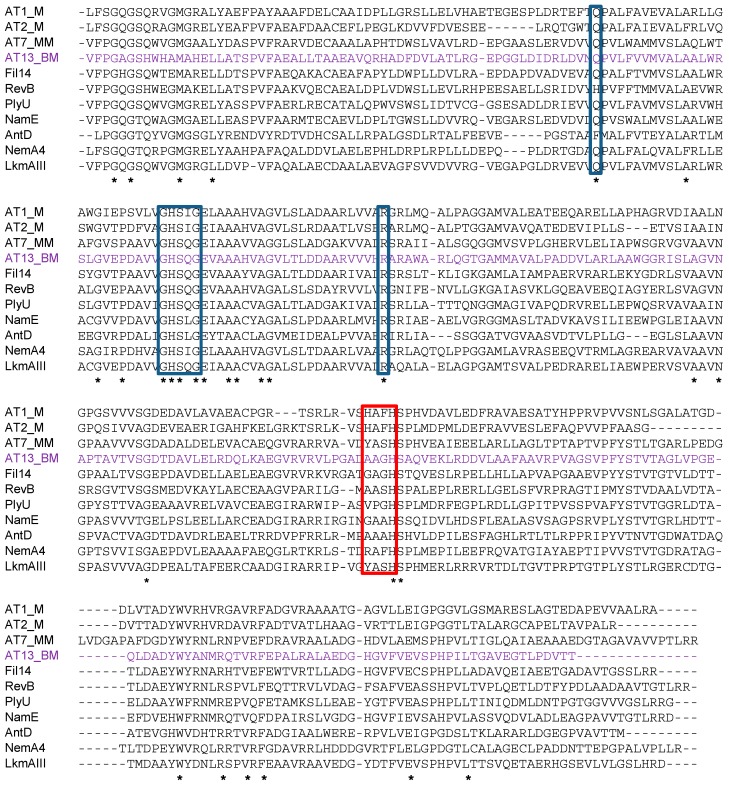
Alignment of acyltransferases with different AT-specificities. AT1-M, AT of thailandin biosynthesis of module 1 with malonyl-CoA specificity; AT2-M, AT of thailandin biosynthesis of module 2 with malonyl-CoA specificity; AT6-MM, AT of thailandin biosynthesis of module 6 with methylmalonyl-CoA specificity; AT13-M, AT of thailandin biosynthesis of module 13 with proposed butylmalonyl-CoA specificity (violet); Fil14, AT of filipin biosynthesis with proposed specificity to hexylmalonyl-CoA; AntD, AT of antimycin biosynthesis with specificity to propanylmalonyl-CoA, butylmalonyl-CoA, pentylmalonyl-CoA and hexylmalonyl-CoA; NemA4, AT of nemadectin biosynthesis with specificity to methyl-propanylmalonyl-CoA and dimethyl-butylmalonyl-CoA; RevB, AT of reveromycin biosynthesis with specificity to butylmalonyl-CoA, isopentylmalonyl-CoA, pentylmalonyl-CoA and hexylmalonyl-CoA; NamE, AT of neoansamycin biosynthesis with specificity to butylmalonyl-CoA and pentylmalonyl-CoA; PlyU, AT of polyoxypeptin biosynthesis with specificity to methyl-butylmalonyl-CoA; motifs highlighted by boxes in blue and red; * indicates highly conserved aa.

The sequences of the described acyltransferases were aligned (Figure A1). The alignment indicates that a later motif may encode for this specificity. Whereas acyltransferases with malonyl-CoA specificity have a HAFH-motif, the sequences differ in the these acyltransferases. The AT13 of the thailandin biosynthesis pathway has a GHSQH- and a AAGH-motif.

Studies on rare actinomycetes such as *Actinokineospora* are very promising in order to identify novel secondary metabolites. The genome of *A. bangkokensis* 44EHW^T^ revealed 34 other secondary metabolite biosynthetic gene clusters indicating that this strain can produce more compounds. In addition, the examination of PKS systems with unusual extender units is important. The knowledge of the specificity of acyltransferases and the underlying sequence motifs gives basics to modify compounds by combinatorial biosynthesis.

## 4. Materials and Methods

### 4.1. Bacterial Growth Condition

*A. bangkokensis* 44EHW^T^ was incubated at 28 °C in TSB medium (CASO Bouillon 30 g/L; Carl Roth GmbH, Karlsruhe, Germany) for 3–5 days, 180 rpm. For production analysis, the strain was pre-cultivated in TSB medium for 2 days at 28 °C and then further cultivated in 100 mL HA medium (0.4% yeast extract, 1% malt extract, and 0.4% glucose; pH 7.4) for 8 days.

### 4.2. Extraction of Secondary Metabolites

After cultivation in HA medium the culture broth was centrifuged (3500× *g*, 10 min, 4 °C). The pH of the supernatant was adjusted to pH 4 by addition of HCl (1 M) and extracted by shaking vigorously with an equal volume of ethyl acetate for 30 min. The organic phase was evaporated to dryness using rotary evaporation at 240 bar. The extract was dissolved in MeOH and analyzed by HPLC/MS.

### 4.3. Analysis of Secondary Metabolite Production by HPLC/MS

The extract was analyzed by a HPLC system equipped with a photodiode array detector (200–600 nm) a mass spectrometer (1100 Series, Agilent Technologies, Waldbronn, Germany) The separation was done by usage of a XBridge™ C18 column (4.6 × 100 mm) with precolumn (4.6 × 20 mm) on a non-linear 0.5% AcOH-CH_3_CN:H_2_O gradient ranged from 20% to 95% at a flow rate of 0.5 mL/min. Thailandin A has UV/vis maxima at 325, 340, and 358 nm and a mass of 754 g/mol, thailandin B has 608 g/mol.

### 4.4. Isolation of Genomic DNA

The strain *A. bangkokensis* 44EHW^T^ was incubated in TSB-Media for 4 days, at 28 °C and 180 rpm. Accordingly, 15 mL of culture was centrifuged (3500× *g*, 10 min, 4 °C) and pellet was washed in 15 mL H_2_O and finally resuspended in 15 mL SET buffer (75 mM NaCl, 25 mM EDTA, 20 mM Tris/HCl pH 8) with lysozyme (100 µg/mL). After incubation for 30 min at 37 °C, RNase (100 µg/mL) was added and further incubated for additional 2 h at 37 °C. Afterwards proteinase K and SDS were added to obtain a final concentration of 100 µg/mL and 0.5%, respectively, and incubated at 50 °C overnight. The DNA was extracted by the addition of equal volume of phenol/chloroform/isoamyl alcohol (25:24:1), inverting carefully for 10 min. After centrifugation (2000× *g*, 20 min, 4 °C), the aqueous phase was transferred into a new tube and the last step was repeated two times. Two volumes of isopropanol (100%) were added to the aqueous phase and genomic DNA was spooled by a glass Pasteur pipette. The DNA was washed in EtOH (70%), dried and finally dissolved in 1 mL H_2_O.

### 4.5. Genome Sequencing of A. bangkokensis 44EHW^T^

The genome of *A. bangkokensis* 44EHW^T^ was sequenced twice. Eurofins Genomics GmbH (Ebersberg, Germany) sequenced the genome using 454 technologies and assembled the reads by Newbler to 247 contigs and 56 scaffolds. In addition, the genome was sequenced at the Center for Biotechnology at the University of Bielefeld, Bielefeld, Germany, using Illumina-HiSeq 1000 technology. Initially, all reads were assembled to a draft genome of 206 contigs and 105 scaffolds using GS de novo assembler version 3.0 (Roche, Branford, CT, USA). The genome was further assembled to 7,453,713 bp genome sequence.

### 4.6. Genome Annotation and Identification of the Thailandin Biosynthetic Gene Cluster

The automatic functional annotation results were obtained using the NCBI prokaryotic genome annotation pipeline [9]. In addition, the ORFs were categorized into different subsystems manually. Secondary metabolite gene clusters were identified by antiSMASH 3.0 [10]. The genes were further analyzed by BLAST [49].

### 4.7. Visualization of the Genome of A. bangkokensis 44EHW^T^

The genome was visualized by Circos, generated with the R package circlize [50,51]. Therefore, the scaffolds with gaps were visualized, the GC content in 500 bp ranges (each 250 bp), the predicted secondary metabolite gene clusters and annotated ORFs grouped by function.

### 4.8. Cloning of Single Crossover Vector pKCLP2_PKS11

For the inactivation of *thaBI* of the PKS cluster #11, a 3087 bp internal fragment of the PKS I gene was amplified using the primers TTATACTGCAGACCGAGGACGAGGTCATC and ATCGGGAGAACTAGACGAACAG by PCR. The PCR product was firstly cloned into pUC19 (Stratagene, La Jolla, CA, USA). Subsequently, it was cut by *Pst*I/*Eco*RV and cloned into the final vector pKCLP2 [52]. For cloning experiments *E. coli* XL1 Blue (Stratagene) was used.

### 4.9. Genetic Manipulation of A. bangkokensis 44EHW^T^

The single crossover vector pKCLP2-PKS11 was transferred into *E. coli* ET12567 (*dam*-, *dcm*-, *hsdM*-, *hsdR*-) [53], which carries the conjugative plasmid pUZ8002. For intergeneric conjugation, *A. bangkokensis* was grown at 28 °C for 2 days in TSB media. The mating mixture was spread on MS agar plates (2% mannitol, 2% soy flour, 2% agarose) containing 10 mM CaCl_2_. The plates were incubated at 28 °C for 16–20 h and overlaid with 1 mL H_2_O containing (0.2 mg phosphomycin and 0.05 mg hygromycin). The plates were further incubated at 28 °C for 6 days. The exconjugants were analyzed for thailandin production.

### 4.10. Alignment of Sequences

The sequences of thailandin acyltransferases and other acyltransferase from biosynthetic pathways with proposed unusual extender units were aligned by Clustal Omega [54] and compared in detail.

## Figures and Tables

**Table 1:** Genome features of *A. bangkokensis* 44EHW^T^.

Feature	Property
total length	7,453,713 bp
GC content	74.1%
number of scaffold	32
number of contigs	79
CDS (total)	6287
genes (coding)	6191
tRNA	50
secondary metabolite biosynthetic gene clusters	35

**Table 2:** Features of the thailandin biosynthetic gene cluster in *A. bangkokensis* 44EHW^T^.

Gene	Protein-ID (PRJNA345323:)	Protein [aa]	Putative Product	Closest Similarity in the Databases (Identity %)
*thaRI*	BJP25_14755	953	LuxR transcriptional regulator	*Streptomyces himastatinicus* (47%)
*thaRII*	BJP25_14760	923	LuxR transcriptional regulator	*Amycolatopsis azurea* (38%)
*thaRIII*	BJP25_14765	921	LuxR transcriptional regulator	*Streptomyces* sp. TAA204 (38%)
*thaRIV*	BJP25_14770	228	LuxR transcriptional regulator	*Allokutzneria albata* (41%)
*thaOI*	BJP25_14775	402	P450 monooxygenase	*Streptomyces* sp. LamerLS-31b (69%)
*thaF*	BJP25_14780	66	ferredoxin	*Streptomyces niger* (73%)
*thaBI*	BJP25_14785	7524	polyketide synthase type I	*Streptomyces* sp. MBT76 (60%)
*thaBII*	BJP25_14790	3501	polyketide synthase type I	*Streptomyces avermitilis* (59%)
*thaBIII*	BJP25_14795	7685	polyketide synthase type I	*Streptomyces* sp. NRRL B-24891 (54%)
*thaBIV*	BJP25_14800	3310	polyketide synthase type I	*Streptomyces avermitilis* (58%)
*thaC*	BJP25_14805	418	crotonyl-CoA carboxylase/reductase	*Streptomyces durhamensis* (71%)
*thaOII*	BJP25_14810	408	P450 monooxygenase	*Streptomyces avermitilis* (65%)
*orf1*	BJP25_14815	295	phosphoesterase PA-phosphatase	*Streptomyces regensis* (70%)
*orf2*	BJP25_14820	297	hypothetical protein	*Blastococcus* sp. URHD0036 (70%)
*orf3*	BJP25_14825	178	hypothetical protein	*Blastococcus* sp. URHD0036 (60%)
*orf4*	BJP25_14830	489	chromosome segregation ATPase	*Kibdelosporangium aridum* (71%)
*orf5*	BJP25_14835	172	hypothetical protein	*Lentzea* sp. DHS C013 (65%)
*orf6*	BJP25_14840	315	hypothetical protein	*Mycobacterium* sp. Root135 (72%)
*orf7*	BJP25_14845	297	oxidoreductase	*Nocardia niigatensis* (63%)
*orf8*	BJP25_14850	312	LuxR transcriptional regulator	*Saccharomonospora* sp. CNQ490 (46%)
*orf9*	BJP25_14855	300	hypothetical protein	*Actinokineospora spheciospongiae* (69%)
*orf10*	BJP25_14860	529	serine protease	*Actinokineospora spheciospongiae* (73%)
*orf11*	BJP25_14865	946	LuxR transcriptional regulator	*Actinokineospora inagensis* (69%)
*orf12*	BJP25_14870	294	LysR transcriptional regulator	*Alloactinosynnema* sp. L-07 (70%)
*thaT*	BJP25_14875	405	MFS transporter	*Blastococcus saxobsidens* (68%)

## Appendix

**Table A1:** Putative secondary metabolite biosynthetic gene clusters in *A. bangkokensis* 44EHW^T^.

Cluster #	Type Secondary Metabolite	Scaffold	from [bp]	to [bp]
1	other	5	222,242	266,147
2	butyrolactone	9	112,144	123,172
3	PKS II/ectoine	9	173,085	217,111
4	nucleosid	10	89,791	111,566
5	bacteriocin	13	79,417	91,126
6	siderophore	13	123,673	135,433
7	*trans* AT-PKS/NRPS/other	14	21,256	106,054
8	NRPS/PKS I	15	12,027	65,701
9	terpene	16	2354	23,460
10	other	17	25,281	68,715
11 *	PKS I *THAILANDIN CLUSTER*	17	179,992	276,120
12	terpene	17	304,613	326,544
13	PKS III	17	319,674	361,110
14	NRPS/ladderane/arylpolyene	17	392,681	462,423
15	terpene	17	454,050	475,513
16 *	oligosaccharide/PKS I	17	557,011	710,221
17	PKS I	17	785,384	815,172
18	PKS I	18	1	35,881
19 *	PKS I/oligosaccharide	19	1	133,613
20	lantipeptide	19	158,947	196,476
21	PKS II	19	200,846	243,937
22	other	19	266,838	310,212
23	PKS I	19	361,716	408,222
24	NRPS/lantipeptide	20	198,722	311,653
25	lassopeptide	22	18,493	40,971
26	lantipeptide	23	93,884	117,519
27	other PKS/PKS I	23	147,485	196,457
28	terpene	23	444,400	466,643
29	butyrolactone	23	494,036	505,109
30	indole	24	1	18,253
31	PKS I	24	37,982	83,864
32	other PKS	24	239,959	308,466
33	NRPS/PKS I	25	71,292	162,064
34	ladderane/NRPS	25	153,235	214,305
35	PKS I	26	1	44,677

* cluster encoding for large PKS I enzymes.

**Table A2:** Identified strains of the genus *Actinokineospora*.

Strain	Product	Genome Sequence *	Literature
*A. auranticolor* IFO 16518	?	-	[55]
*A. baliensis* ID03-0561^T^	?	-	[56]
*A. bangkokensis* 44EHW^T^	thailandins A and B	this study MKQR01000000	[7,8]
*A. cianjurensis* ID03-0810^T^	?	-	[56]
*A. cibodasensis* ID03-0784^T^	?	-	[56]
*A. diospyrosa* NRRL B-24047	?	-	[57]
*A. enzanensis* IFO 16517	?	GCA_000374445.1	[55]
*A. fastidiosa* NRRL B-16697	macrobicyclic peptide antibiotic	-	[58,59,60]
*A. globicatena* NRRL B-24048	?	-	[57]
*A. guangxiensis* GK-6T	?	-	[61]
*A. inagensis* NRRL B-24050	?	GCA_000482865.1	[57]
*A. mzabensis* PAL84	?	-	[62]
*A. riparia* IFO 14541	compound with antimycoplasmic activity	-	[63]
*A. soli* YIM 75948T	?	-	[64]
*A. spheciospongiae* EG49	actinosporins A–D, products from co-cultivation	GCA_000564855.1	[6,39,40,41,65,66]
*A. terrae* IFO 15668	?	-	[57]

* GenBank assembly accession number.

## References

[1] Gomez-Escribano J.P., Bibb M.J. (2014). Heterologous expression of natural product biosynthetic gene clusters in *Streptomyces coelicolor*: From genome mining to manipulation of biosynthetic pathways. J. Ind. Microbiol. Biotechnol..

[2] Bode H.B., Bethe B., Höfs R., Zeeck A. (2002). Big effects from small changes: possible ways to explore nature’s chemical diversity. Chembiochem.

[3] Gao C., Hindra, Mulder D., Yin C., Elliot M.A. (2012). Crp is a global regulator of antibiotic production in *Streptomyces*. MBio.

[4] Gessner A., Heitzler T., Zhang S., Klaus C., Murillo R., Zhao H., Vanner S., Zechel D.L., Bechthold A. (2015). Changing biosynthetic profiles by expressing *bldA* in *Streptomyces* strains. ChemBioChem.

[5] Hasegawa T. (1988). *Actinokineospora*: A new genus of the Actinomycetales. Actinomycetologica.

[6] Harjes J., Ryu T., Abdelmohsen U.R., Moitinho-Silva L., Horn H., Ravasi T., Hentschel U. (2014). Draft genome sequence of the antitrypanosomally active sponge-associated bacterium *Actinokineospora* sp. strain EG49. Genome Announc..

[7] Intra B., Matsumoto A., Inahashi Y., Omura S., Takahashi Y., Panbangred W. (2013). *Actinokineospora bangkokensis* sp. nov., isolated from rhizospheric soil. Int. J. Syst. Evol. Microbiol..

[8] Intra B., Greule A., Bechthold A., Euanorasetr J., Paululat T., Panbangred W. (2016). Thailandins A and B, new polyene macrolactone compounds isolated from *Actinokineospora bangkokensis* strain 44EHW^T^, possessing antifungal activity against anthracnose fungi and pathogenic yeasts. J. Agric. Food Chem..

[9] Tatusova T., DiCuccio M., Badretdin A., Chetvernin V., Nawrocki E.P., Zaslavsky L., Lomsadze A., Pruitt K.D., Borodovsky M., Ostell J. (2016). NCBI prokaryotic genome annotation pipeline. Nucleic Acids Res..

[10] Weber T., Blin K., Duddela S., Krug D., Kim H.U., Bruccoleri R., Lee S.Y., Fischbach M.A., Müller R., Wohlleben W. (2015). antiSMASH 3.0—A comprehensive resource for the genome mining of biosynthetic gene clusters. Nucleic Acids Res..

[11] Yadav G., Gokhale R.S., Mohanty D. (2003). Computational approach for prediction of domain organization and substrate specificity of modular polyketide synthases. J. Mol. Biol..

[12] Bisang C., Long P.F., Corte´s J., Westcott J., Crosby J., Matharu A.-L., Cox R.J., Simpson T.J., Staunton J., Leadlay P.F. (1999). A chain initiation factor common to both modular and aromatic polyketide synthases. Nature.

[13] Erb T.J., Berg I.A., Brecht V., Müller M., Fuchs G., Alber B.E. (2007). Synthesis of C5-dicarboxylic acids from C2-units involving crotonyl-CoA carboxylase/reductase: the ethylmalonyl-CoA pathway. Proc. Natl. Acad. Sci. USA.

[14] Erb T.J., Brecht V., Fuchs G., Müller M., Alber B.E. (2009). Carboxylation mechanism and stereochemistry of crotonyl-CoA carboxylase/reductase, a carboxylating enoyl-thioester reductase. Proc. Natl. Acad. Sci. USA.

[15] Quade N., Huo L., Rachid S., Heinz D.W., Müller R. (2012). Unusual carbon fixation gives rise to diverse polyketide extender units. Nat. Chem. Biol..

[16] Sandy M., Rui Z., Gallagher J., Zhang W. (2012). Enzymatic synthesis of dilactone scaffold of antimycins. ACS Chem. Biol..

[17] Wilson M.C., Moore B.S. (2012). Beyond ethylmalonyl-CoA: The functional role of crotonyl-CoA-carboxylase/reductase homologs in expanding polyketide diversity. Nat. Prod. Rep..

[18] Keatinge-Clay A. (2008). Crystal structure of the erythromycin polyketide synthase dehydratase. J. Mol. Biol..

[19] Caffrey P., Lynch S., Flood E., Finnan S., Oliynyk M. (2001). Amphotericin biosynthesis in *Streptomyces nodosus*: deductions from analysis of polyketide synthase and late genes. Chem. Biol..

[20] Fjærvik E., Zotchev S.B. (2005). Biosynthesis of the polyene macrolide antibiotic nystatin in *Streptomyces noursei*. Appl. Microbiol. Biotechnol..

[21] Caffrey P. (2003). Conserved amino acid residues correlating with ketoreductase stereospecificity in modular polyketide synthases. ChemBioChem.

[22] Keatinge-Clay A.T., Stroud R.M. (2006). The structure of a ketoreductase determines the organization of the β-carbon processing enzymes of modular polyketide synthases. Structure.

[23] Bonnett S.A., Whicher J.R., Papireddy K., Florova G., Smith J.L., Reynolds K.A. (2013). Structural and stereochemical analysis of a modular polyketide synthase ketoreductase domain required for the generation of a *cis*-alkene. Chem. Biol..

[24] Pöplau P., Frank S., Morinaka B.I., Piel J. (2013). An enzymatic domain for the formation of cyclic ethers in complex polyketides. Angew. Chem. Int. Ed..

[25] Berkhan G., Hahn F. (2014). A dehydratase domain in ambruticin biosynthesis displays additional activity as a pyran-forming cyclase. Angew. Chem. Int. Ed..

[26] Luhavaya H., Dias M.V.B., Williams S.R., Hong H., de Oliveira L.G., Leadlay P.F. (2015). Enzymology of pyran ring A formation in salinomycin biosynthesis. Angew. Chem. Int. Ed..

[27] Kamiński D.M. (2014). Recent progress in the study of the interactions of amphotericin B with cholesterol and ergosterol in lipid environments. Eur. Biophys. J..

[28] Fanos V., Cataldi L. (2000). Amphotericin B-induced nephrotoxicity: A review. J. Chemother..

[29] Tevyashova A.N., Olsufyeva E.N., Solovieva S.E., Printsevskaya S.S., Reznikova M.I., Trenin A.S., Galatenko O.A., Treshalin I.D., Pereverzeva E.R., Mirchink E.P. (2013). Structure-antifungal activity relationships of polyene antibiotics of the amphotericin B group. Antimicrob. Agents Chemother..

[30] Brautaset T., Sletta H., Nedal A., Borgos S.E.F., Degnes K.F., Bakke I., Volokhan O., Sekurova O.N., Treshalin I.D., Mirchink E.P. (2008). Improved antifungal polyene macrolides via engineering of the nystatin biosynthetic genes in *Streptomyces noursei*. Chem. Biol..

[31] Chen S., Huang X., Zhou X., Bai L., He J., Jeong K.J., Lee S.Y., Deng Z. (2003). Organizational and mutational analysis of a complete FR-008/candicidin gene cluster encoding a structurally related polyene complex. Chem. Biol..

[32] Palacios D.S., Dailey I., Siebert D.M., Wilcock B.C., Burke M.D. (2011). Synthesis-enabled functional group deletions reveal key underpinnings of amphotericin B ion channel and antifungal activities. Proc. Natl. Acad. Sci. USA.

[33] Gary-Bobo C.M. (1989). Polyen-sterol interaction and selective toxicity. Biochimie.

[34] Cybulska B., Bolard J., Seksek O., Czerwinski A., Borowski E. (1995). Identification of the structural elements of amphotericin B and other polyene macrolide antibiotics of the hepteane group influencing the ionic selectivity of the permeability pathways formed in the red cell membrane. Biochim. Biophys. Acta.

[35] Pandey R.C., Narasimhachari N., Rinehart K.L., Millington D.S. (1972). Polyene antibiotics. IV. Structure of chainin. J. Am. Chem. Soc..

[36] Ceder O., Ryhage R. (1964). The structure of filipin. Acta Chem. Scand..

[37] Shih H.-D., Liu Y.-C., Hsu F.-L., Mulabagal V., Dodda R., Huang J.-W. (2003). Fungichromin: A substance from *Streptomyces padanus* with inhibitory effects on *Rhizoctonia solani*. J. Agric. Food Chem..

[38] Wang Y.-F., Wei S.-J., Zhang Z.-P., Zhan T.-H., Tu G.-Q. (2012). Antifungalmycin, an antifungal macrolide from *Streptomyces padanus* 702. Nat. Products Bioprospect..

[39] Abdelmohsen U., Cheng C., Viegelmann C., Zhang T., Grkovic T., Ahmed S., Quinn R., Hentschel U., Edrada-Ebel R. (2014). Dereplication strategies for targeted isolation of new antitrypanosomal actinosporins A and B from a marine sponge associated-*Actinokineospora* sp. EG49. Mar. Drugs.

[40] Grkovic T., Abdelmohsen U.R., Othman E.M., Stopper H., Edrada-Ebel R., Hentschel U., Quinn R.J. (2014). Two new antioxidant actinosporin analogues from the calcium alginate beads culture of sponge-associated *Actinokineospora* sp. strain EG49. Bioorg. Med. Chem. Lett..

[41] Dashti Y., Grkovic T., Abdelmohsen U., Hentschel U., Quinn R. (2014). Production of induced secondary metabolites by a co-culture of sponge-associated actinomycetes, *Actinokineospora* sp. EG49 and *Nocardiopsis* sp. RV163. Mar. Drugs.

[42] Wang X.-K., Jin J.-L. (2014). Crucial factor for increasing the conjugation frequency in *Streptomyces netropsis* SD-07 and other strains. FEMS Microbiol. Lett..

[43] Aparicio J.F., Caffrey P., Gil J.A., Zotchev S.B. (2003). Polyene antibiotic biosynthesis gene clusters. Appl. Microbiol. Biotechnol..

[44] Takahashi S., Toyoda A., Sekiyama Y., Takagi H., Nogawa T., Uramoto M., Suzuki R., Koshino H., Kumano T., Panthee S. (2011). Reveromycin A biosynthesis uses RevG and RevJ for stereospecific spiroacetal formation. Nat. Chem. Biol..

[45] Li S., Li Y., Lu C., Zhang J., Zhu J., Wang H., Shen Y. (2015). Activating a cryptic ansamycin biosynthetic gene cluster to produce three new naphthalenic octaketide ansamycins with *n*-pentyl and *n*-butyl side chains. Org. Lett..

[46] Seipke R.F., Patrick E., Hutchings M.I. (2014). Regulation of antimycin biosynthesis by the orphan ECF RNA polymerase sigma factor σ AntA. PeerJ.

[47] Carter G.T., Nietsche J.A., Hertz M.R., Williams D.R., Siegel M.M., Morton G.O., James J.C., Borders D.B. (1988). LL-F28249 antibiotic complex: A new family of antiparasitic macrocyclic lactones. Isolation, characterization and structures of LL-F28249 α, β, δ, λ. J. Antibiot. (Tokyo).

[48] Du Y., Wang Y., Huang T., Tao M., Deng Z., Lin S. (2014). Identification and characterization of the biosynthetic gene cluster of polyoxypeptin A, a potent apoptosis inducer. BMC Microbiol..

[49] Madden T. (2003). The BLAST Sequence Analysis Tool. The NCBI Handbook.

[50] Krzywinski M., Schein J., Birol I., Connors J., Gascoyne R., Horsman D., Jones S.J., Marra M.A. (2009). Circos: An information aesthetic for comparative genomics. Genome Res..

[51] Gu Z., Gu L., Eils R., Schlesner M., Brors B. (2014). Circlize implements and enhances circular visualization in R. Bioinformatics.

[52] Petzke L., Bechthold A. (2010). Transgenese in Streptomyceten: Transposons, Rekombinasen und Meganukleasen. Ph.D. Thesis.

[53] MacNeil D.J., Gewain K.M., Ruby C.L., Dezeny G., Gibbons P.H., MacNeil T. (1992). Analysis of *Streptomyces avermitilis* genes required for avermectin biosynthesis utilizing a novel integration vector. Gene.

[54] Sievers F., Wilm A., Dineen D., Gibson T.J., Karplus K., Li W., Lopez R., McWilliam H., Remmert M., Söding J. (2011). Fast, scalable generation of high-quality protein multiple sequence alignments using Clustal Omega. Mol. Syst. Biol..

[55] Otoguro M., Hayakawa M., Yamazaki T., Tamura T., Hatano K., Iimura Y. (2001). Numerical phenetic and phylogenetic analyses of *Actinokineospora* isolates, with a description of *Actinokineospora auranticolor* sp. nov. and *Actinokineospora enzanensis* sp. nov.. Actinomycetologica.

[56] Lisdiyanti P., Otoguro M., Ratnakomala S., Lestari Y., Hastuti R.D., Triana E., Katsuhiko A., Widyastuti Y. (2010). *Actinokineospora baliensis* sp. nov., *Actinokineospora cibodasensis* sp. nov. and *Actinokineospora cianjurensis* sp. nov., isolated from soil and plant litter. Int. J. Syst. Evol. Microbiol..

[57] Tamura T., Hayakawa M., Nonomura H., Yokota A., Hatano K. (1995). Four new species of the genus *Actinokineospora*: *Actinokineospora inagensis* sp. nov., *Actinokineospora globicatena* sp. nov., *Actinokineospora terrae* sp. nov., and *Actinokineospora diospyrosa* sp. nov.. Int. J. Syst. Bacteriol..

[58] Celmer W.D., Cullen W.P., Moppett C.E., Routien J.B., Shibakawa R., Tone J. (1977). Antibiotics Produced by Species of *Pseudonocardia*. U.S. Patent.

[59] Henssen A., Kothe H.W., Kroppenstedt R.M. (1987). Transfer of *Pseudonocardia azurea* and *Pseudonocardia fastidiosa* to the genus *Amycolatopsis*, with emended species description. Int. J. Syst. Bacteriol..

[60] Labeda D.P., Price N.P., Tan G.Y.A., Goodfellow M., Klenk H.-P. (2010). Emended description of the genus *Actinokineospora* (Hasegawa 1988) and transfer of *Amycolatopsis fastidiosa* (Henssen et al. 1987) as *Actinokineospora*
*fastidiosa* comb. nov.. Int. J. Syst. Evol. Microbiol..

[61] Wu H., Liu B. (2015). *Actinokineospora guangxiensis* sp. nov., isolated from soil. Int. J. Syst. Evol. Microbiol..

[62] Aouiche A., Bouras N., Mokrane S., Zitouni A., Schumann P., Spröer C., Sabaou N., Klenk H.-P. (2015). *Actinokineospora mzabensis* sp. nov., a novel actinomycete isolated from Saharan soil. Antonie Van Leeuwenhoek.

[63] Hasegawa T. (1991). Studies on motile arthrospore-bearing rare actinomycetes. Actinomycetologica.

[64] Tang X., Zhou Y., Zhang J., Ming H., Nie G.-X., Yang L.-L., Tang S.-K., Li W.-J. (2012). *Actinokineospora soli* sp. nov., a thermotolerant actinomycete isolated from soil, and emended description of the genus *Actinokineospora*. Int. J. Syst. Evol. Microbiol..

[65] Abdelmohsen U.R., Pimentel-Elardo S.M., Hanora A., Radwan M., Abou-El-Ela S.H., Ahmed S., Hentschel U. (2010). Isolation, phylogenetic analysis and anti-infective activity screening of marine sponge-associated actinomycetes. Mar. Drugs.

[66] Kämpfer P., Glaeser S.P., Busse H.-J., Abdelmohsen U.R., Ahmed S., Hentschel U. (2015). *Actinokineospora spheciospongiae* sp. nov., isolated from the marine sponge *Spheciospongia vagabunda*. Int. J. Syst. Evol. Microbiol..

